# The Combining Ability for Grain Yield and Some Related Characteristics in Rice (*Oryza sativa* L.) Under Normal and Water Stress Conditions

**DOI:** 10.3389/fpls.2022.866742

**Published:** 2022-07-08

**Authors:** Mohamed S. Abd El-Aty, Youssef S. Katta, Abd El Moaty B. El-Abd, Samiha M. Mahmoud, Omar M. Ibrahim, Mohamed A. Eweda, Mohamed T. El-Saadony, Synan F. AbuQamar, Khaled A. El-Tarabily, Amira M. El-Tahan

**Affiliations:** ^1^Department of Agronomy, Faculty of Agriculture, Kafr El Sheikh University, Kafr El-Sheikh, Egypt; ^2^Rice Research and Training Center, Agricultural Research Center, Field Crops Research Institute, Kafr El-Sheikh, Egypt; ^3^Department of Plant Production, Arid Lands Cultivation Research Institute, The City of Scientific Research and Technological Applications, SRTA-City, Alexandria, Egypt; ^4^Department of Agricultural Microbiology, Faculty of Agriculture, Zagazig University, Zagazig, Egypt; ^5^Department of Biology, College of Science, United Arab Emirates University, Al-Ain, United Arab Emirates; ^6^Khalifa Center for Genetic Engineering and Biotechnology, United Arab Emirates University, Al-Ain, United Arab Emirates; ^7^Harry Butler Institute, Murdoch University, Murdoch, WA, Australia

**Keywords:** combining ability, earliness, heterosis, rice genotypes, water stress conditions

## Abstract

Drought is considered a major threat to rice production. This study aimed to determine the effects of drought stress on the estimates of heterosis and the combining ability of rice genotypes for the number of days to 50% heading, plant height, number of panicles per plant, panicle length, number of filled grains per panicle, and grain yield per plant. Field experiments were conducted at the Rice Research and Training Center, Kafr El Sheikh, Egypt, during the rice-growing season in 2018 and 2019. Eight rice genotypes (Giza178, Giza179, Sakha106, Sakha107, Sakha108, WAB1573, NERICA4, and IET1444) were crossed in a half-diallel cross in the rice-growing season in 2018, which yielded a wide range of variability in numerous agronomic traits and drought tolerance measurements. In 2019, these parents and their 28 F_1_ crosses were produced by employing a three-replication randomized complete block design under normal and water stress conditions. The results showed remarkable differences across the studied genotypes under normal and water stress conditions. Under both conditions, Sakha107 was the best general combiner for earliness and short stature. Giza179 and Sakha108 were the best general combiners for grain yield per plant and one or more of its characteristics. Furthermore, in both normal and water stress conditions, Giza179 exhibited the highest general combining ability effects for all attributes that were evaluated. Under normal and water stress conditions, the Giza179 × Sakha107 cross demonstrated substantial and desirable specific combining ability effects on all the examined traits, which suggested that it could be considered for use in rice hybrid breeding programs. Therefore, we recommend that these vital indirect selection criteria to be considered for improving rice grain yield under drought conditions.

## Introduction

Rice (*Oryza sativa* L.) is a well-known crop that is consumed by most of the world’s population. It has a low fat content and high carbohydrate, protein, vitamin, and mineral content. Rice is widely grown in many parts of the world, including Egypt (Ullah Zaid et al., 2018; El-Mowafi et al., 2021; Abd El-Aty et al., 2022a). The global population is estimated to increase from 7.7 billion today to 9 billion in 2035. Therefore, there would be an increase in global rice demand from 763 million tons to 850 million tons. However, in the past decade, only a 1% annual increase in rice yield was reported, and this average was the highest among rice-producing countries. Rice consumption continues to increase with the increase in global population (Kumar, 2018; Gaballah et al., 2022).

In contrast, scarcity of irrigation water is a major stumbling block to improve rice production worldwide (El-Mowafi et al., 2021; Abd El-Aty et al., 2022b). Water stress is a critical limiting factor during the early stages of rice development and establishment, which affects both stem elongation and leaf area expansion during growth. In the past three decades, rice sector in Egypt has outperformed that in the rest of the world in terms of rice production and yield (Sari et al., 2020; Gaballah et al., 2022). Rice yield per hectare increased from 5.7 t ha^–1^ in the 1980s to 9.52 t ha^–1^ in the 2000s as a result of the widespread adoption of semi-dwarf and early maturing Egyptian cultivars. However, the rice production could not keep up with the growing population and diminishing water resources (Dianga et al., 2020).

Using the hybrid rice technology that employs heterosis is an effective approach for enhancing the rice yield. This method utilizes heterosis, which refers to the superiority of an F_1_ hybrid over its parents. Compared with the traditional high-yielding inbred varieties, the F_1_ hybrid rice has a 15%–20% yield advantage (Sari et al., 2020). Identifying good parental lines to develop hybrid combinations is crucial so that hybrid rice technology is effective. As a result, rice breeders continuously select suitable parental lines (Suvi et al., 2021). The combining ability has been employed to understand the potential of a given parental line to pass on its genetic information to its descendants to overcome this challenge. The general combining ability (GCA) indicates an additive gene action and measures the average performance of parental lines. The specific combining ability (SCA) represents a non-additive gene action associated with dominance, overdominance, and epistatic effects and quantifies the performance of hybrid combinations (Dianga et al., 2020).

In hybrid combinations, genetic variations in parental lines have a remarkable impact. Many morphological and molecular techniques have been used to assess the genetic variability of parental lines utilized in hybrid rice breeding. Although the biological basis for drought resistance is unknown, several plant breeding initiatives have focused on selecting genotypes with better output in drought-prone areas. Any selection or hybridization breeding programs for developing drought-tolerant varieties require precise estimates of genetic variance components for the traits of interest, including additive, dominant, and non-allelic interaction effects (Ullah Zaid et al., 2018; Suvi et al., 2021).

At present, utilizing heterosis in self-pollinated crops, particularly in rice, is impossible. Furthermore, heterosis management requires the ability of genotypes in hybrids to mix in general and specialized ways. Plant breeders can use the diallel analysis to determine a breeding system that can be adopted and breeding materials that possess the best likelihood of achieving success in selection. This strategy, which was initially adopted by Griffing (1956) can be used for several crop to carry out self-pollination.

The present study aimed to identify optimum cultivars and cross combinations by assessing five native and three “alien” rice genotypes and their F_1_ diallel crosses. Thus, the potential for heterosis expression was determined for a set of agronomic and grain yield-related variables to evaluate the combining ability effects and gene action modes in the inheritance of grain yield-related agronomic traits.

## Materials and Methods

This study was conducted at an experimental farm of the Rice Research and Training Center, Sakha, Kafr Elsheikh, Egypt, during the rice-growing seasons in 2018 and 2019. Eight rice cultivars were chosen to reflect various agronomic traits and drought resistance measurements (five local varieties: Giza178, Giza179, Sakha106, Sakha107, and Sakha108 and three exotic varieties: WAB1573, NERICA4, and IET1444). Rice cultivar grains at 140 kg ha^–1^ were prepared for sowing in the nursery. Sowing was performed in late April 2018 and 2019. Table 1 lists the names, origins, and other agronomic characteristics of these parents.

**TABLE 1:** Origin and main characteristics of the eight rice genotypes used as parents in the half-diallel cross.

No	Genotype	Parentage	Origin	Grain shape	Variety group	Drought tolerance
1	Giza178	Giza175/Milyang49	Egypt	Medium	Indica/Japonica	Moderate
2	Giza179	GZ6296-12/GZ1368-5-S-4	Egypt	Short	Indica/Japonica	Tolerant
3	Sakha106	Giza177/Hexi30	Egypt	Short	Japonica	Sensitive
4	Sakha107	Giza177/BL1	Egypt	Short	Japonica	Sensitive
5	Sakha108	Sakha101/HR5824/Sakha101	Egypt	Short	Japonica	Sensitive
6	IET	TN1 × CO29	India	Short	Indica/Japonica	Tolerant
7	WAB1573	Introduced	Côte d’Ivoire	Long	Indica	Tolerant
8	NERICA4	CG14/WAB56-104	Africa rice center	Long	Indica	Tolerant

A permanent field was prepared, as recommended by the Ministry of Agriculture, Egypt. Calcium superphosphate (15.5% P_2_O_5_) was applied at a rate of 238 kg ha^–1^ during soil tillage. Then, 25-day-old seedlings were transplanted in a 20 × 20 cm^2^ field, with 4–5 seedlings hill^–1^. Potassium sulfate (48% K_2_O) was added at a concentration of 58 kg K_2_O ha^–1^ and divided into two equal doses at 15 and 35 days after transplantation (DAT). Nitrogen fertilizers in the form of urea (46% N) at a concentration of 165 kg ha^–1^ were also added and divided into three equal doses at 15, 35, and 55 DAT.

During the 2018 rice-growing season, the parents were crossed in 8 × 8 diallel crosses, eliminating reciprocals, yielding 28 crosses. In the 2019 season, the parents and their F_1_ hybrid seeds were sown in a dry seedbed, and 30-day-old seedlings were transplanted individually into field plots in two separate irrigating experiments. A well-watered condition was maintained using continuous flooding every 4 days, with an adequate submersion depth to ensure that all surface areas were covered by water in each irrigation incident. A water-deficit treatment was maintained by the application of irrigation water every 10 days without standing water. A stress condition was applied 15 days after the transplantation date and until maturity. A flow meter was used to measure the applied irrigation quantities for each treatment, which were 13,090 and 8330 m^3^ ha^–1^ under well-watered and water-deficit conditions, respectively.

Water stress treatment was performed at 10 DAT. Two trials were set up in a three-replication randomized complete block design. Each replicate had five rows of parents and three rows of F_1_ hybrids. The row was 5-m long, with a spacing of 20 × 20 cm^2^ between rows of seedlings.

The number of days to 50% heading was recorded at the heading stage. In contrast, the plant height, number of panicles per plant, panicle length (cm), number of filled grains per panicle, and grain yield per plant (g) at maturity of 25 randomly chosen single plants from each entry were recorded.

For each attribute, heterosis was calculated based on parents vs. crosses sum of squares by partitioning the sum of squares of the genotype to its components. All characters were subjected to analysis of variance, as described by Steel et al. (1997), using IRRISTAT and R software version 4.1.0 2021. The obtained mean values were compared using the least significant difference. GCA and SCA were analyzed using method 11, model 1 according to the techniques of Griffing (1956).

Supplementary Figure 1 shows the weather data (rain in mm, average temperature in °C, relative humidity in %, and radiation in MJ m^2^) recorded in 2018 and 2019, which were obtained from https://power.larc.nasa.gov.

Chemical and mechanical analyses of soil and organic matter were performed according to Page et al. (1982) at the Agricultural Research Center, Ministry of Agricultural, Egypt. Some chemical and physical properties of the soil present in the experimental site at a depth of 0–30 cm are presented in Table 2.

**TABLE 2:** Some physical and chemical properties of the experimental soil before sowing at the two locations.

Soil properties	Value
**Mechanical:**	
Clay (%)	56.00
Silt (%)	32.00
Sand (%)	12.00
Texture	Clayey
**Chemical:**	
Organic matter (%)	1.50
pH (1:2.5 soil suspension)	8.44
Ec (ds m^–1^)	3.34
Total N (ppm)	430.50
Available P (ppm)	12.00
Available K (ppm)	432
**Soluble anions (meq L^–1^):**	
HCO^–^_3_	6.20
Cl^–^	9.10
**Soluble cations (meq L^–1^):**	
Ca^++^	10.70
Mg^++^	5.00
Na^++^	2.00
K^+^	15.60
Total carbonate (%)	14.00

Twenty-two stress tolerance indices were calculated for all the genotypes based on grain yield under non-stress and stress conditions. The names, equations, and references of the stress tolerance indices are listed in Supplementary Table 1.

## Results

### Analysis of Variance

Table 3 shows the analysis of variance for yield and its components under drought and regular watering conditions. For all the traits studied under drought and regular irrigation conditions, the mean squares of the genotypes and their partitions, parents, crosses, and parents vs. crosses, were significantly different, indicating the presence of diversity to a large extent among all the genotypes used in this study. These results indicated that the rice genotypes responded differently to stress and non-stress conditions.

**TABLE 3:** Mean square estimates of ordinary analysis and combining ability analysis for yield and related traits under normal and water stress conditions.

SOV	df	Number of days to 50% heading (day)		Plant height (cm)		Number of panicle per plants	
				
		N	D	N	D	N	D
Replications	2	2.06	0.01	6.04	0.23	0.42	0.65
Genotypes	35	148.38**	171.24**	359.20**	333.85**	261.15**	68.52**
Parents	7	161.31**	172.61**	114.54**	159.00**	200.74**	33.69**
Crosses	27	150.46**	177.23**	367.94**	383.18**	285.75**	79.91**
Parents vs. Crosses	1	1.65	0.00	1835.84**	226.06**	19.86**	4.91**
Error	70	1.46	0.93	3.57	1.58	5.69	1.31
GCA	7	212.82**	227.83**	165.73**	330.21**	323.38**	85.74**
SCA	28	8.62**	14.39**	108.23**	56.55**	27.97**	7.11**
Error term	70	0.49	0.31	1.19	0.53	1.90	0.44
GCA/SCA		2.611	1.616	0.154	0.588	1.233	1.277

**SOV**	**df**	**Panicle length (cm)**		**Number of filled grains per panicle**		**Grain yield per plant (g)**	
				
		**N**	**D**	**N**	**D**	**N**	**D**

Replications	2	13.85	0.05	102.72	0.15	0.66	4.73
Genotypes	35	173.16**	74.47**	2609.70**	2083.67**	113.29**	41.28**
Parents	7	23.37**	20.50**	3918.01**	2055.40**	66.08**	16.36**
Crosses	27	209.31**	76.75**	2194.94**	1862.59**	120.87**	46.47**
Parents vs. Crosses	1	245.93**	390.59**	4650.19**	8250.68**	238.97**	75.56**
Error	70	7.35	1.13	92.56	5.85	0.21	0.94
GCA	7	117.86**	64.36**	3370.35**	2466.83**	135.34**	40.67**
SCA	28	42.69**	14.94**	244.79**	251.49**	13.37**	7.03**
Error term	70	2.45	0.38	30.85	1.95	0.07	0.31
GCA/SCA		0.287	0.439	1.561	0.988	1.017	0.601

** indicates significance at 0.01 probability level. GCA, general combining ability; SCA, specific combining ability; SOV, sources of variation; df, degrees of freedom; N, normal condition; D, water stress condition.

For all the measured traits, both GCA and SCA mean squares were highly significant. This finding highlights the significance of additive and non-additive genetic factors in influencing these traits and consequently the performance of the rice genotypes.

### Mean Performance

The mean performances of the parents and F_1_ hybrids for the traits studied under drought and regular irrigation conditions are shown in Table 4.

**TABLE 4:** Mean performance of parents and their F_1_ generation in a half-diallel cross for yield and some related traits under normal and water stress conditions.

No.	Genotypes	Number of days to 50% heading (day)		Plant height (cm)		Number of panicles per plant	
				
		N	D	N	D	N	D
1	Giza178	105.00	99.00	102.28	86.67	42.87	25.83
2	Giza179	98.67	92.33	127.67	88.80	46.00	27.67
3	Sakha106	104.00	109.00	113.28	94.00	32.00	22.00
4	Sakha107	98.67	91.33	107.53	90.67	35.73	29.33
5	Sakha108	112.00	108.67	106.00	84.00	30.00	23.67
6	IET1444	99.00	93.33	112.33	85.33	40.33	32.33
7	WAB1573	108.33	105.67	109.50	93.00	32.00	30.07
8	NERICA 4	108.00	105.02	134.87	123.84	24.47	23.67
9	Giza178 × Giza179	92.67	88.67	97.60	82.33	34.87	26.80
10	Giza178 × Sakha106	107.00	109.00	100.00	80.00	36.00	25.33
11	Giza178 × Sakha107	89.00	86.67	104.40	82.67	34.00	29.00
12	Giza178 × Sakha108	102.00	103.33	110.60	80.33	27.07	22.33
13	Giza178 × IET1444	93.00	88.33	108.40	86.00	25.00	23.65
14	Giza178 × WAB1573	108.33	104.67	103.87	96.20	26.10	24.00
15	Giza178 × NERICA4	101.67	98.67	124.40	93.67	22.33	22.00
16	Giza179 × Sakha106	106.00	102.00	108.80	90.87	25.53	18.67
17	Giza179 × Sakha107	95.67	96.67	121.67	91.67	25.00	22.00
18	Giza179 × Sakha108	114.33	111.00	112.73	87.00	23.00	19.00
19	Giza179 × IET1444	101.00	94.33	136.00	98.67	32.00	27.00
20	Giza179 × WAB1573	116.00	108.00	125.33	101.00	20.00	18.33
21	Giza179 × NERICA4	107.00	105.33	122.78	98.33	18.73	17.00
22	Sakha106 × Sakha107	95.00	91.00	114.47	103.53	38.07	25.67
23	Sakha106 × Sakha108	98.00	93.33	106.33	87.00	30.00	24.00
24	Sakha106 × IET1444	94.00	88.67	118.33	92.00	39.00	35.33
25	Sakha106 × WAB1573	103.00	96.67	139.33	116.67	26.33	24.00
26	Sakha106 × NERICA4	101.00	91.33	116.67	110.33	20.00	18.67
27	Sakha107 × Sakha108	112.00	106.00	98.33	86.33	27.00	22.33
28	Saakha107 × IET1444	109.00	98.67	113.67	88.67	35.00	30.00
29	Sakha107 × WAB1573	118.00	108.67	109.67	101.00	20.00	18.00
30	Sakha107 × NERICA4	111.00	106.67	99.33	87.33	19.00	17.00
31	Sakha108 × IET1444	100.33	96.00	106.73	88.00	37.13	28.00
32	Sakha108 × WAB1573	104.00	95.00	114.67	103.67	31.33	27.67
33	Sakha108 × NERICA4	102.00	93.33	116.33	105.67	19.00	19.00
34	IET1444 × WAB1573	113.00	111.00	111.73	99.00	23.37	23.00
35	IET1444 × NERICA4	109.67	108.33	134.57	115.67	22.00	19.00
36	WAB1573 × NEICA4	106.00	104.00	109.37	96.33	20.00	19.90
L.S.D 0.05		1.98	1.58	3.08	2.05	3.89	1.88
L.S.D 0.01		2.63	2.10	4.10	2.73	5.17	2.50

**No.**	**Genotypes**	**Panicle length (cm)**		**Number of filled grains per panicle**		**Grain yield per plant**	
				
		**N**	**D**	**N**	**D**	**N**	**D**

1	Giza178	26.40	24.10	103.00	98.00	44.53	34.67
2	Giza179	27.43	25.68	177.17	161.67	46.83	37.67
3	Sakha106	22.30	20.00	112.67	104.67	41.83	32.33
4	Sakha107	27.47	26.40	118.33	101.00	47.30	38.33
5	Sakha108	27.10	23.67	146.00	129.00	52.03	40.00
6	IET1444	28.83	26.33	163.67	152.67	46.80	36.67
7	WAB1573	22.30	20.27	180.00	162.00	36.93	30.00
8	NERICA 4	26.53	23.67	151.13	147.33	40.30	35.67
9	Giza178 × Giza179	25.90	22.61	112.67	101.00	45.90	36.00
10	Giza178 × Sakha106	24.67	23.48	98.33	94.33	50.73	38.33
11	Giza178 × Sakha107	25.13	22.33	115.33	105.00	53.20	40.33
12	Giza178 × Sakha108	25.18	22.00	142.67	132.00	54.70	41.33
13	Giza178 × IET1444	25.33	23.00	163.22	155.00	45.10	38.00
14	Giza178 × WAB1573	25.30	24.00	143.33	141.67	42.97	30.00
15	Giza178 × NERICA4	25.30	22.00	155.33	147.00	44.87	33.00
16	Giza179 × Sakha106	20.23	16.67	86.33	81.00	40.90	31.00
17	Giza179 × Sakha107	25.00	22.00	90.00	87.00	44.77	32.00
18	Giza179 × Sakha108	23.00	19.00	115.67	112.33	46.77	35.00
19	Giza179 × IET1444	25.07	23.00	109.00	98.00	43.30	33.67
20	Giza179 × WAB1573	20.00	18.67	132.67	131.00	33.87	28.33
21	Giza179 × NERICA4	18.73	17.00	142.33	138.67	36.33	30.33
22	Sakha106 × Sakha107	20.13	17.33	94.00	87.00	42.33	33.00
23	Sakha106 × Sakha108	27.67	24.33	122.00	110.33	49.37	37.67
24	Sakha106 × IET1444	28.00	26.00	134.33	122.00	50.83	37.67
25	Sakha106 × WAB1573	26.00	24.67	152.00	138.00	37.87	32.33
26	Sakha106 × NERICA4	20.00	18.67	164.67	153.33	35.83	30.67
27	Sakha107 × Sakha108	23.33	18.33	132.40	118.67	44.07	30.00
28	Saakha107 × IET1444	25.00	23.00	168.33	154.67	55.97	38.33
29	Sakha107 × WAB1573	20.00	18.00	175.33	161.00	44.57	35.33
30	Sakha107 × NERICA4	19.00	17.67	178.67	165.00	42.03	31.67
31	Sakha108 × IET1444	24.69	21.00	153.60	134.67	38.80	34.00
32	Sakha108 × WAB1573	29.33	27.00	155.33	143.00	40.90	32.33
33	sakha108 × NERICA4	21.00	19.00	162.00	153.33	35.83	28.33
34	IET1444 × WAB1573	19.67	18.33	171.87	142.00	32.70	29.67
35	IET1444 × NERICA4	24.00	23.67	180.67	175.33	33.63	28.67
36	WAB1573 × NEICA4	20.80	19.33	180.97	150.33	35.13	31.00
L.S.D 0.05		1.53	146	15.66	3.93	0.77	1.56
L.S.D 0.01		2.03	1.94	20.83	5.22	0.99	2.07

*N, normal condition; D, water stress condition.*

The number of days to 50% heading of the parental varieties Sakha107, Giza179, and IET1444 were 98.67, 98.67, and 99.00 days under regular irrigation and 92.23, 91.33, and 93.33 days under stress conditions, respectively. Thus, these were the earliest varieties under regular irrigation. Meanwhile, Sakha107, Giza179, and IET1444 were the earliest varieties under water stress conditions, with 91.33, 92.33, and 93.33 days to 50% heading, respectively.

For the number of panicles per plant, the parental varieties Giza179, Giza178, and IET1444 exhibited the highest mean values of 46, 42.87, and 40.33 panicles per plant under regular irrigation and 27.67, 25.83, and 32.33 panicles per plant under stress conditions, respectively. In contrast, NERICA4 and Sakha106 had the lowest values of 24.47 panicles per plant under normal conditions and 22.00 panicles per plant under stress conditions, respectively.

The parental variety of IET1444 had the tallest panicle length (28.83 and 26.33 cm) compared with the other parents under normal and stress conditions, respectively. For the number of filled grains per panicle, the parental varieties Giza179 and WAB1573 exhibited the highest mean values of 177.17 and 180 under normal and stress conditions, respectively. The parental varieties Sakha107 and Sakha108 had the highest grain yield per plant of 47.30 and 52.03 g per plant under normal and stress conditions, respectively.

Seven crosses (Giza178 × Sakha107, Giza178 × Giza179, Giza178 × IET1444, Giza179 × Sakha107, Sakha106 × IET1444, Sakha106 × Sakha107, and Sakha106 × Sakha108) were earlier combinations than their parental mean values under normal and stress conditions, while five crosses (Giza178 × Sakha107, Giza178 × IET1444, Sakha106 × IET1444, Giza178 × Giza179, and Sakha106 × Sakha107) were earlier than their parental lines. Regarding plant height, the mean performances of the crosses varied significantly. Three crosses (Giza178 × Giza179, Sakha107 × Sakha108, and Sakha107 × NERICA4) out of 28 were shorter than their parents under normal and stress conditions. These crosses produced high grain yield and were resistant to lodging. The cross that had the highest mean number of panicles per plant (39 and 35.33 panicles per plant under normal and stress conditions, respectively) was Sakha106 × IET1444.

Sakha108 × WAB1573 had the highest panicle length (29.33 and 27.00 cm under normal and stress conditions, respectively). Furthermore, WAB1573 × NEICA4 and IET1444 × NERICA4 had a higher number of filled grains per panicle under normal conditions, whereas IET1444 × NERICA4 and Sakha107 × NERICA4 had a higher number of filled grains per panicle under stress conditions.

### Combining Ability

The mean square values of the GCA and SCA effects are provided in Table 3. Under both conditions, the data revealed extremely significant GCA and SCA estimates for all the traits studied. Both types of combining abilities appeared to contribute to the inheritance of these characteristics. Under both conditions, the GCA/SCA ratios exceeded unity for the number of days to 50% heading (day), panicles per plant, number of filled grains per panicle, and grain yield per plant (g), indicating that additive gene action is more important than non-additive gene action in controlling these features.

#### General Combining Ability

Table 5 shows the impacts of GCAs on the number of days to 50% heading. The parental varieties Giza179, Sakha107, and IET1444 exhibited desirable significant adverse GCA effects for the number of days to 50% heading under both conditions. These adverse GCA effects suggested that these parents were the strongest general combiners for earliness. In contrast, the parents Giza178, Giza179, Sakha108, and IET1444 demonstrated highly significant negative GCA effects under stress conditions, whereas Sakha106 and IET1444 were deemed as the best donors for earliness.

**TABLE 5:** Estimates of general combining ability effects for yield and some related traits for parental genotypes under normal and water stress conditions.

No.	Genotypes	Number of days to 50% heading (day)		Plant height (cm)		Number of panicles per plant	
				
		N	D	N	D	N	D
1	Giza178	0.28	0.59**	−0.92**	−1.61**	6.62**	2.51
2	Giza179	−5.08**	−3.71**	−5.04**	−7.67**	2.62**	1.23
3	Sakha106	2.12**	3.99**	2.45*	−1.67**	−2.23**	−2.73
4	Sakha107	−6.65**	−7.08**	1.83**	2.90**	2.61**	1.84
5	Sakha108	5.25**	4.49**	−6.99**	−6.11**	−2.20**	−1.66
6	IET1444	−3.32**	−5.38**	0.83*	−1.31**	3.59**	3.57
7	WAB1573	5.75**	5.16**	3.55**	7.61**	−3.56**	−0.82
8	NERICA 4	1.65**	1.93**	4.28**	7.84**	−7.45**	−3.92
S.E (g_i_)		0.21	0.16	0.32	0.21	0.41	0.20
S.E (g_l_-g_j_)		0.31	0.25	0.49	0.32	0.61	0.30
L.S.D 0.05		0.41	0.33	0.65	0.43	0.81	0.39
L.S.D 0.01		0.55	0.44	0.86	0.57	1.08	0.52

**No.**	**Genotypes**	**Panicle length**		**Number of filled grains per panicle**		**Grain yield per plant (g)**	
				
		**N**	**D**	**N**	**D**	**N**	**D**

1	Giza178	1.83**	1.87**	−1.63	−1.79**	1.11**	1.42**
2	Giza179	1.37**	1.22**	−5.05**	−3.36**	4.02**	2.48**
3	Sakha106	−1.72**	−1.91**	−29.79**	−24.43**	−1.06**	−1.38**
4	Sakha107	0.31*	0.35*	−18.66	−18.16**	1.39**	0.92**
5	Sakha108	−0.29*	−1.12	4.22*	2.94**	4.36**	1.35**
6	IET1444	1.55**	1.38	9.18**	7.58**	0.64**	0.72**
7	WAB1573	−1.02**	−0.26	19.18**	16.38**	−5.38**	−2.95**
8	NERICA4	−2.03**	−1.52**	22.55**	20.84**	−5.09**	−2.55**
S.E (g_i_)		0.16	0.15	1.64	0.41	0.08	0.17
S.E (g_l_-g_j_)		0.24	0.23	2.48	0.62	0.12	0.25
L.S.D 0.05		0.32	0.31	3.28	0.8	0.16	0.33
L.S.D 0.01		0.43	0.41	4.38	1.09	0.21	0.44

* and ** indicate significance at 0.05 and 0.01 probability levels, respectively. N, normal condition; D, water stress condition.

The parents Giza178, Giza179, Sakha107, and IET1444 exhibited highly significant positive GCA effects for the number of panicles per plant under normal conditions. Under both conditions, the parents Giza178, Giza179, Sakha107, and IET1444 exhibited beneficial significant positive GCA effects. In addition, the genotypes Sakha108, IET1444, WAB1573, and NERICA4 showed highly significant positive GCA effects under both conditions. All genotypes showed highly significant positive GCA effects under both conditions for grain yield per plant, with the exception of Sakha106, WAB1573, and NERICA4. This finding could be useful for rice breeding programs that intend to generate cultivars having a high yield.

#### Specific Combining Ability

SCA effects for the crosses can estimate the non-additive impact. Estimates of SCAs of F_1_ hybrids are presented in Supplementary Table 2. The SCA for the number of days to 50% heading (day) was negative and highly significant in eight crosses under normal and stress conditions, indicating that one or more combinations could assist in the selection of early maturing parental lines of rice. Six crosses showed adverse and highly significant desirable SCA effects for plant height under both conditions. Only two crosses exhibited positive and highly significant desirable SCA effects for the number of panicles per plant under both conditions (Giza178 × Giza179 and Sakha108 × IET1444). For panicle length, 12 crosses showed desirable SCA effects under both conditions. Nine out of the 28 cross combinations showed positive and highly significant desirable SCA effects for the number of filled grains per panicle under both normal and stress conditions. Furthermore, the estimates of SCA effects for grain yield were positive and highly significant in eight crosses under both normal and stress conditions.

#### Drought Stress Tolerance

Stress indices can be used to quantify the stress response based on the crop yield. These are more readily usable owing to their more straightforward interpretation compared with raw yield data. Many drought tolerance indices have been proposed (Supplementary Table 1) to assess stress-tolerant genotypes using mathematical equations, describing the relationship between yields under stress and non-stress conditions. These indices can be classified into two categories: the first includes indices with maximum values that indicate high-stress tolerance, while the other category includes indices with minimum values that indicate high-stress tolerance. The use of these indices will assist in the identification of stable, high-yield, and drought-tolerant genotypes (Sofi et al., 2018). Owing to the long labels of crosses, an abbreviation was used to present the results and figures (Table 6).

**TABLE 6:** Abbreviations of genotypes.

G1	Giza178	G19	Giza179 × IET1444
G2	Giza179	G20	Giza179 × WAB1573
G3	Sakha106	G21	Giza179 × NERICA4
G4	Sakha107	G22	Sakha106 × Sakha107
G5	Sakha108	G23	Sakha106 × Sakha108
G6	IET1444	G24	Sakha106 × IET1444
G7	WAB1573	G25	Sakha106 × WAB1573
G8	NERICA4	G26	Sakha106 × NERICA4
G9	Giza178 × Giza179	G27	Sakha107 × Sakha108
G10	Giza178 × Sakha106	G28	Saakha107 × IET1444
G11	Giza178 × Sakha107	G29	Sakha107 × WAB1573
G12	Giza178 × Sakha108	G30	Sakha107 × NERICA4
G13	Giza178 × IET1444	G31	Sakha108 × IET1444
G14	Giza178 × WAB1573	G32	Sakha108 × WAB1573
G15	Giza178 × NERICA4	G33	Sakha108 × NERICA4
G16	Giza179 × Sakha106	G34	IET1444 × WAB1573
G17	Giza179 × Sakha107	G35	IET1444 × NERICA4
G18	Giza179 × Sakha108	G36	WAB1573 × NEICA4

Supplementary Table 3 presents 22 stress indices and yield values under stress and non-stress conditions. The first 14 indices are maximum value indices, where the maximum values indicates tolerance. Simultaneously, the last eight indices are minimum value indices, where the minimum value indicates tolerance. Supplementary Table 4 presents the yield ranks under stress and non-stress conditions, ranks of the 22 stress indices, and the average of these ranks.

**FIGURE 1:**
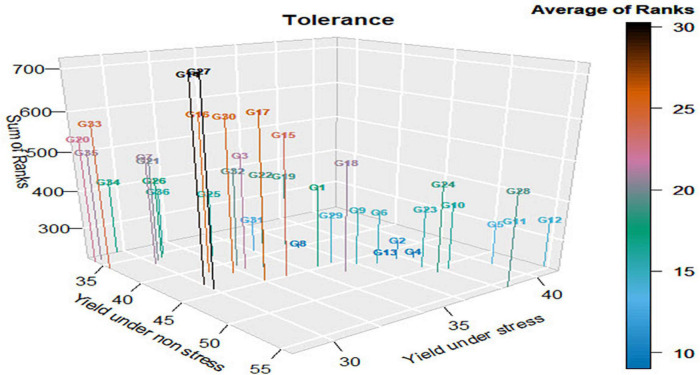
Tolerance of genotypes according to the average rank of 22 stress indices (small number of average ranks signifies tolerance). All Gs correspond to the genotype identified in Table 6.

The results in Table 6 and Supplementary Table 3 revealed that G13 (Giza178 × IET1444) was the most tolerant genotype, with an average rank (AR) of nine (Figure 1); however, G14 (Giza178 × WAB1573) and G27 (Sakha107 × Sakha108) were the least tolerant genotypes (AR, 30). Both G8 (NERICA4) and G4 (Sakha107) exhibited a similar intolerance, with an AR of 10, and were the second and third tolerant genotypes. G2 (Giza179) was the fourth tolerant genotype (AR, 11). As the average rank values increased, the tolerance of genotypes decreased, as shown in Supplementary Table 3. Taking the average ranks of the different indices was helpful because of the other results of the indices. This is obvious for the rank of G34 (IET1444 × WAB1573), with an AR of 17, as 11 indices ranked this genotype first as the most tolerant genotype.

These indices included yield stability index, relative stress index, golden mean, tolerance index, stress susceptibility index, stress susceptibility percentage index, yield reduction, abiotic stress tolerance index, mean productivity index, Schnieders stress susceptibility index, and sensitivity drought index. These indices were primarily driven by the difference in yield between stress and non-stress conditions; however, it did not consider the yield of the G34 genotype relative to that of the other genotypes under stress and non-stress conditions. In contrast, the indices like mean productivity, geometric mean productivity, harmonic mean, stress tolerance index, yield index, modified stress tolerance index-I, modified stress tolerance index-II, relative efficiency index, and mean relative performance ranked G34 significantly lower or as the lowest tolerant genotype because most of them considered the yield of G34 relative to that of the other genotypes under stress and non-stress conditions.

G28 (Saakha107 × IET1444) ranked first based on yield under non-stress conditions; however, its AR was 19 and occupied the 21st position. Moreover, G12 (Giza178 × Sakha108) was ranked first based on the yield under stress conditions; however, its average rank was 14 and occupied the eighth position. These results ensure that the ranking of the genotypes depends on the amount of reduction in yield or the difference between the yields under stress and non-stress conditions, in this order.

### Correlation Analysis

**FIGURE 2:**
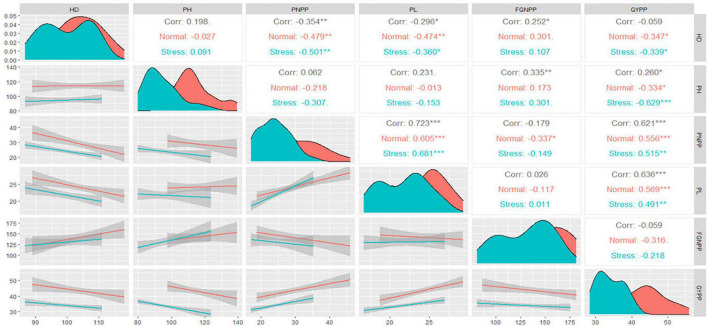
Spearman correlation matrix for the traits studied under normal and water stress conditions. HD, number of days to 50% heading; PH, plant height; PNPP, panicle number per plant; PL, panicle length; FGNPP, number of filled grains per panicle; GYPP, grain yield per plant. ^*^, ^**^ and ^***^ indicate significance at 0.05, 0.01 and 0.001 probability levels, respectively.

The upper triangle in Figure 2 shows the Spearman coefficient correlation matrix between each pair of the studied traits under normal and water stress conditions. Both plant height and the number of days to 50% heading (HD) showed a negative and significant correlation with grain yield per plant under normal and water stress conditions. Meanwhile, the number of filled grains per panicle showed a negative and insignificant correlation with grain yield per plant. Both panicle number per plant and panicle length showed a positive and significant correlation with grain yield per plant under normal and water stress conditions. Subjecting rice to water stress did not change the relationship between grain yield per plant and the other traits. In contrast, water stress weakened the relationship between panicle number per plant and filled grains per panicle, wherein it was significant under normal conditions and not significant under water stress conditions. This change was caused by the decrease in panicle number per plant under water stress conditions, as illustrated in the density plots in the diagonal of Figure 2, where the peak of panicle number per plant under water stress conditions corresponded to the lower value on the *x*-axis.

The diagonal in Figure 2, illustrates the density plots of the traits studied using the smoothed function of the values. The highest density of the values was indicated by the area under the curve and the peak of the density plot. From the density plots, it was observed that all the traits studied had different peaks under normal and water stress conditions. Thus, the values of these traits under normal conditions are concentrated at higher values compared to those under water stress conditions. However, a remarkable overlap was observed in panicle length and HD values, i.e., the water stress effects were not stable on these traits. The density plots thus showed that the effects of water stress were mainly on both the magnitude of the traits and their density.

### Cluster Analysis

**FIGURE 3:**
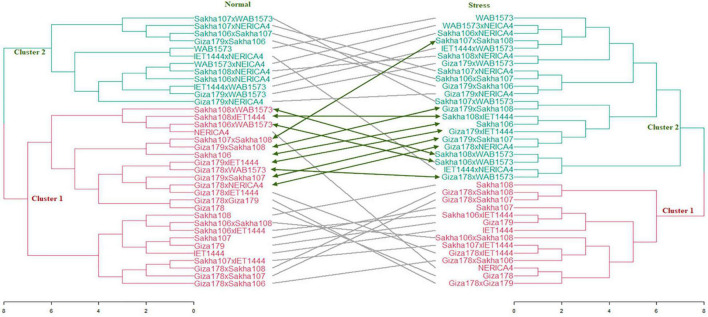
A tangle-gram showing results of cluster analysis based on Euclidean coefficient and Ward method under normal and water stress conditions.

To cluster the genotypes under normal and water stress conditions, cluster analysis was performed using Euclidean as a distance measure of dissimilarity and Ward’s algorithm on R software version 4.1.0 2021. Grain yield per plant and panicle length values were used to construct a distance matrix and generate a tangle-gram to show the similarities among all the genotypes under normal and water stress conditions (Figure 3). The data were standardized owing to their different scales. Because the results of Fuzzy C-Means showed low overlap between clusters, hard clustering methods were used to construct the tangle-gram (Figure 3). Six methods were compared using agglomerative coefficients to choose the most efficient method for clustering the data. These methods were average, generalized average, single, weighted, complete, and Ward. The agglomerative coefficients were 0.81, 0.88, 0.71, 0.84, 0.89, and 0.94, respectively, under normal conditions and 0.86, 0.90, 0.64, 0.86, 0.91, and 0.95, respectively, under water stress conditions.

**FIGURE 4:**
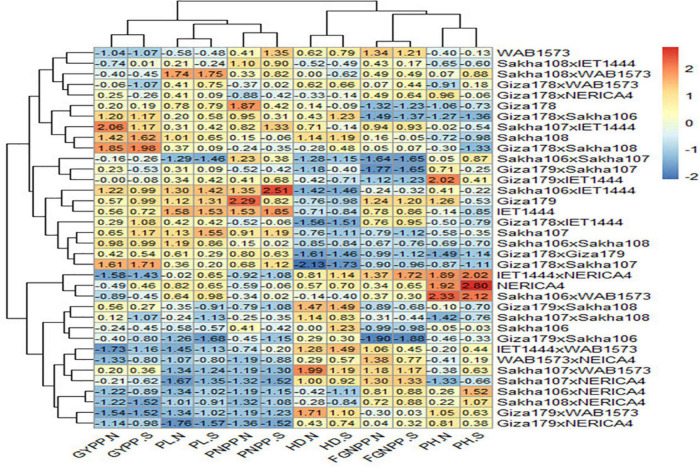
A heatmap of the relationship between genotypes and the traits studied under normal (.N) and water stress (.S) conditions. GYPP, grain yield per plant; PL, panicle length; PNPP, panicle number per plant; HD, number of days to 50% heading; FGNPP, number of filled grains per panicle; PH, plant height.

Based on Figure 3, all the genotypes were grouped into two clusters under normal and water stress conditions, and the average of the traits studied is summarized in Table 7. The structure of the clusters remained the same when the genotypes were subjected to water stress conditions, with the exception of the Sakha108 × WAB1573, Sakha108 × IET1444, Sakha106 × WAB1573, Giza179 × Sakha108, Sakha106, Giza179 × IET1444, Giza178 × WAB1573, Giza179 × Sakha107, Giza178 × NERICA4, and Sakha107 × Sakha108 genotypes, which moved from cluster 1 under normal conditions to cluster 2 under water stress conditions because they were less stable than the other members of their cluster. Overall, a heatmap was generated to determine the relationship between genotypes and the studied traits under normal and water stress conditions (Figure 4).

**TABLE 7:** Average of the traits studied for the two clusters under normal and water stress conditions.

Condition	Cluster	HD	PH	PNPP	PL	FGNPP	GYPP
Normal	Cluster1	100.59	111.44	33.76	25.39	130.76	46.33
	Cluster2	108.73	117.29	22.09	22.18	155.94	39.12
Water stress	Cluster1	95.25	88.02	26.94	23.31	119.02	36.92
	Cluster2	103.70	99.99	21.23	20.31	140.30	31.35

*HD, number of days to 50% heading; PH, plant height; PNPP, panicle number per plant; PL, panicle length; FGNPP, number of filled grains per panicle; GYPP, grain yield per plant.*

## Discussion

Choosing proper parents and crosses is a challenge for breeders of high-yielding rice varieties with improved grain qualities. The hybrid combining abilities of parental lines of rice can be an efficient tool to improve their rice production. Combining ability analysis is one of the most practical approaches to estimate its effects for the selection of desired parents and crosses (El-Mowafi et al., 2015; Akanksha and Jaiswal, 2019). Moreover, the combining ability was investigated to identify the best genetic potential for developing cross combinations with desirable characteristics and to observe the genetic impact involved in trait expression (Sprague and Tatum, 1942).

For all the traits tested, the results of combining ability showed that both GCA and SCA mean squares were extremely significant. This finding emphasizes the relevance of both additive and non-additive genetic factors in determining how these traits perform under normal and stress conditions. Under normal and stress conditions, the GCA/SCA ratios were more significant than unity for the number of days to 50% heading (day), number of panicles per plant, number of filled grains per panicle, and grain yield per plant (g) under regular irrigation conditions. The SCA estimation of the hybrid combinations demonstrated that all the hybrids showed significantly positive SCA effects for at least one yield characteristic.

The analysis of variance of several genotypes under different conditions (drought and non-drought) at both sites revealed significant differences for all the characteristics assessed, implying that the germplasm utilized in the study possessed substantial genetic diversity. Thus, the studied genotypes can improve grain yield and other agronomic traits in drought-prone crops.

Similar results were found in other studies (Ganapati et al., 2020; Abd El-Aty et al., 2022a). However, under deficit irrigation, non-additive gene effects, appear to be highly important for plant height, panicle length, number of filled grains per panicle, and grain yield per plant, as shown by Akanksha and Jaiswal (2019). Thus, additive gene effects significantly contribute to the inheritance of these qualities, and a pedigree technique of selection can be used to improve them. Such results demonstrated the role of the cumulative effects of additive × additive interactions of positive alleles. Some previous studies reported the effects of additive gene action on yield quality and quantity traits (El-Hity et al., 2015; El-Mowafi et al., 2018; Herwibawa et al., 2019). Simultaneously, other studies revealed the effects of additive and non-additive genes and their benefits on developing hybrid rice varieties (Sreeramachandra et al., 2000; Huang et al., 2015).

For traits regulated by non-additive gene activities, hybridization followed by selection in subsequent generations may be performed. The earliest parents were the Sakha107, Giza179, and IET1444 varieties with 98.66, 98.67, and 99.00 days under regular irrigation and 92.23, 91.33, and 93.33 days under stress conditions, respectively. Under both conditions, the parental varieties Giza179 and WAB1573 showed the most significant mean number of filled grains per panicle. Under normal and stressful conditions, the parental varieties Sakha107 and Sakha108 exhibited the highest grain yield per plant. Vanaja et al. (2006) used a 6 × 6 half-diallel cross to evaluate the combining ability of rice yield and yield components. They proposed that additive and non-additive gene effects are essential in determining the yield and the components showing the most yield (Mandal and Roy, 2001; Kusaka et al., 2005; Moradi et al., 2012; El-Malky and Al-Daej, 2018; Manjunath et al., 2020).

Because the Giza179 × Sakha107 cross showed highly significant desirable SCA effects for all the studied traits under normal and water stress conditions, they could be recommended for use in rice hybrid breeding programs. Earlier findings have also confirmed these results (El-Mowafi et al., 2015; Manjunath et al., 2020).

The cubic clustering criterion (Milligan and Cooper, 1985) was used to identify whether there were clusters in the data. Fuzzy C-Means is a soft clustering algorithm (Bezdek, 1973, 1981) and was used to determine if overlapping existed between the clusters. The Ward method had the highest coefficient compared with the other five methods under normal and water stress conditions; thus, it was chosen for cluster analysis. To validate the optimum number of clusters in the data, internal validation was performed, where voting among 30 indices was used to determine the relevant number of clusters in the data (Ward, 1963; Charrad et al., 2014).

## Conclusion

Under normal and water stress conditions, Sakha107 was the best general combiner for earliness and short stature. Giza179 and Sakha108 were the best general combiners for grain yield per plant and one or more of its characteristics. Furthermore, in both normal and water stress conditions, Giza179 displayed the highest GCA impacts for all attributes evaluated. In addition, under normal and water stress conditions, the Giza179 × Sakha107 cross demonstrated highly substantial and desirable SCA effects on all the examined traits.

## Data Availability Statement

The original contributions presented in this study are included in the article/Supplementary Material, further inquiries can be directed to the corresponding author/s.

## Author Contributions

MA, YK, SA, and KE-T conceived and designed the research. MA, SA, KE-T, and AE-T supervised the study and wrote the manuscript. MA, YK, AE-A, SM, OI, and AE-T performed field experiments. MA, YK, AE-A, SM, OI, and ME developed the biochemical and physiological analyses. MA, ME-S, SA, KE-T, and AE-T analyzed the data. YK, AE-A, SM, OI, ME, and ME-S assisted with experiments and/or data evaluation. All authors critically revised the manuscript and approved the final version.

## Conflict of Interest

The authors declare that the research was conducted in the absence of any commercial or financial relationships that could be construed as a potential conflict of interest.

## Publisher’s Note

All claims expressed in this article are solely those of the authors and do not necessarily represent those of their affiliated organizations, or those of the publisher, the editors and the reviewers. Any product that may be evaluated in this article, or claim that may be made by its manufacturer, is not guaranteed or endorsed by the publisher.
