# Supplementary material for: The Combining Ability for Grain Yield and Some Related Characteristics in Rice (Oryza sativa L.) Under Normal and Water Stress Conditions
Source: Front Plant Sci. 2022 Jul 8;13:866742. doi: 10.3389/fpls.2022.866742 (PMC9305714; doi:10.3389/fpls.2022.866742)
Supplement: Supplementary file 1 [file Data_Sheet_1.pdf]

Supplementary material

The Combining Ability for Grain Yield and Some Related Characteristics in Rice (*Oryza sativa* L.) Under Normal and Water Stress Conditions

Mohamed S. Abd El-Aty, Youssef S. Katta, Abd El Moaty B. El-Abd, Samiha M. Mahmoud, Omar M. Ibrahim, Mohamed A. Eweda, Mohamed T. El-Saadony, Synan F. AbuQamar*, Khaled A. El-Tarabily* and Amira M. El-Tahan

*** Correspondence:**
Synan AbuQamar: sabuqamar@uaeu.ac.ae
Khaled El-Tarabily: ktarabily@uaeu.ac.ae

Supplementary material

Supplementary Figure S1. Weather data for 2018 and 2019 seasons.

Supplementary Table S1. Stress screening indices based on yield.

Supplementary Table S2. Estimates of specific combining ability effects for yield and related traits in F_1 crosses under normal and water stress conditions.

Supplementary Table S3. Values of 22 stress screening indices based on grain yield under normal or water stress conditions.

Supplementary Table S4. Rank of genotypes by 22 indices and yield under normal or water stress along with their average rank.

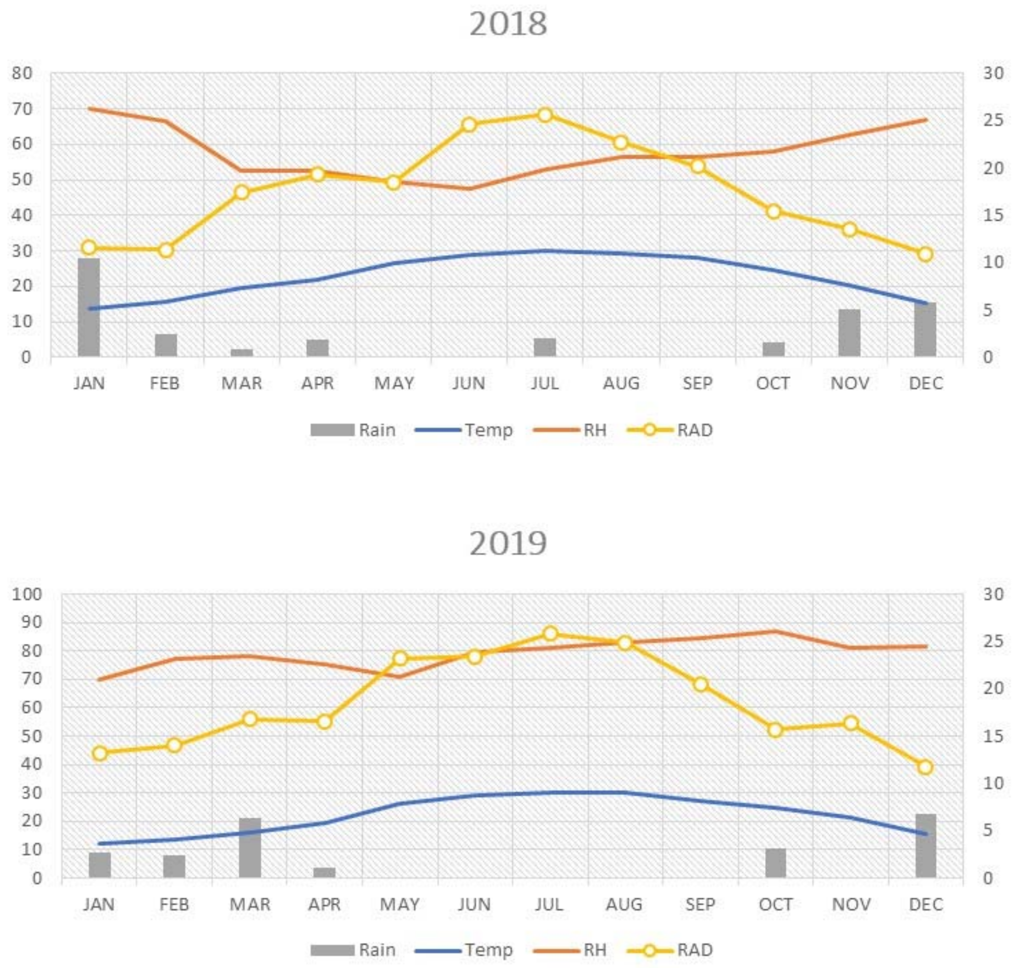

Supplementary Figure S1. Weather data for 2018 and 2019 seasons.

Supplementary Table S1. Stress screening indices based on yield.

Index	Formula	Reference
Indices with maximum values corresponding to more tolerance to water-deficit stress		
Mean productivity (MP)<br>Geometric mean productivity (GMP)<br>Harmonic mean (HM)<br>Stress Tolerance Index (STI)<br>Yield index (YI)<br>Modified stress tolerance index-I<br>(MSTI1)<br>Modified stress tolerance index- II<br>(MSTI2)<br>Yield stability index (YSI)<br>Relative stress index (RSI)<br>Drought index (DI)<br>Stress/non-stress productivity index<br>(SNPI)<br>Relative efficiency index (REI)<br>Mean relative performance (MRP)<br>Golden mean (Gm)	$(Y_S + Y_{NS}) / 2$<br>$(Y_{NS})^{(1/2)} * Y_S$<br>$2 * (Y_S * Y_{NS}) / (Y_S + Y_{NS})$<br>$(Y_s * Y_{NS}) / (Y_{NS\cdot m})^2$<br>$Y_s / Y_{S\cdot m}$<br>$((Y_{NS})^2 / (Y_{NS.m})^2) *((Y_s * Y_{NS}) / (Y_{NS\cdot m})^2)$<br>$((Y_s)^2 / (Y_{S\cdot m})^2) *((Y_s * Y_{NS}) / (Y_{NS\cdot m})^2)$<br>$Y_s / Y_{NS}$<br>$(Y_s / Y_{NS}) / (Y_{S\cdot m} / Y_{NS.m})$<br>$(Y_s*(Y_s / Y_{NS})) / Y_{S.m}$<br>$((Y_{NS}+Y_s) / (Y_{NS}-Y_s))^{(1/3)} * (Y_{NS}* Y_s$<br>$*Y_s)^{(1/3)}$<br>$(Y_s*Y_{NS})/(Y_{S\cdot m}*Y_{NS.m})$<br>$(Y_s / Y_{S\cdot m}) + (Y_{NS} / Y_{NS.m})$<br>$(Y_{NS} + Y_s) / (Y_{NS} - Y_s)$	Rosielle and Hamblin (1981)<br>Fernandez (1992)<br>Bidinger et al. (1987)<br>Fernandez (1992)<br>Gavuzzi et al. (1997)<br>Farshadfar and Sutka (2002)<br>Farshadfar and Sutka (2002)<br>Bouslama and Schapaugh Jr (1984)<br>Fischer and Wood (1979)<br>Bidinger et al. (1987)<br>Mousavi et al. (2008)<br>Ramirez-Vallejo and Kelly (1998)<br>Ramirez-Vallejo and Kelly (1998)<br>Moradi et al. (2012)
Indices with minimum values corresponding to more tolerance to water-deficit stress		
Tolerance index (TOL)<br>Stress Susceptibility Index (SSI)<br>Stress Susceptibility Percentage Index<br>(SSPI)<br>Yield Reduction (YR)<br>Abiotic Stress Tolerance Index (ATI)<br>Mean Productivity Index (MPI)<br>Schnieders Stress Susceptibility Index<br>(SSSI)<br>Sensitivity Drought Index (SDI)	$Y_{NS} – Y_s$<br>$(1 - (Y_s / Y_{NS})) / (1 - (Y_{S\cdot m} / Y_{NS.m}))$<br>$(Y_{NS} - Ys) / (2 * Y_{NS.m})$<br>$1- (Ys / Y_{NS})$<br>$((Y_{NS} - Ys) / (Y_{NS.m} / Y_{S\cdot m})) * (Y_{NS} * Ys)^{(1/2)}$<br>$(Y_{NS} - Ys)/2$<br>$1-(Ys / Y_{NS}) - (1- (Y_{S\cdot m} / Y_{NS.m}))$<br>$(Y_{NS} -Ys)/ Y_{NS}$	Rosielle and Hamblin (1981)<br>Schneider et al. (1997)<br>Mousavi et al. (2008)<br>Choukan et al. (2006)<br>Mousavi et al. (2008)<br>Rosielle and Hamblin (1981)<br>Schneider et al. (1997)<br>Farshadfar and Javadinia (2011)

Y_S, yield under water stress; Y_{NS}, yield under normal conditions; $Y_{S.m}$, means of yield under water stress; $Y_{NS.m}$, mean of yield under normal conditions.

Supplementary Table S2. Estimates of specific combining ability effects for yield and related traits in F_1 crosses under normal and water stress conditions.

No.	Genotypes	Number of days to 50% heading (day)		Plant height (cm)		Number of panicles per plant	
		N	D	N	D	N	D
1	Giza178 × Giza179	-0.51	-4.26**	19.74**	3.74**	7.87**	0.01
2	Giza178 × Sakha106	-2.38**	4.70**	-2.13**	2.93**	-1.29	-1.70**
3	Giza178 × Sakha107	1.05	-1.90**	-7.26**	-4.97**	-2.40*	1.07*
4	Giza178 × Sakha108	2.49**	3.87**	0.03	-2.63**	-3.32**	-1.10*
5	Giza178 × IET1444	-1.95**	-1.60**	-1.46	-6.09**	1.23	2.33**
6	Giza178 × WAB1573	-1.68**	0.20	-7.01**	-7.35**	0.04	4.46**
7	Giza178 × NERICA4	2.09**	2.79**	17.63**	23.26**	-3.60**	1.15*
8	Giza178 × Giza179	-8.35**	-11.33**	-13.69**	-2.67**	5.58**	4.38**
9	Giza179 × Sakha107	-3.25**	-2.26**	-6.27**	-6.90**	-0.13	2.01**
10	Giza179 × Sakha108	-2.15**	2.84**	8.75**	-0.23	-2.25*	-1.15*
11	Giza179 × IET1444	-2.58**	-2.30**	-1.27	0.64	-10.10**	-5.07**
12	Giza179 × WAB1573	3.69**	3.50**	-8.53**	1.92**	-1.86	-0.33
13	Giza179 × NERICA4	1.12*	0.74	11.28**	-0.85	-1.73	0.77
14	Sakha106 × Sakha107	-3.78**	0.04	3.51**	-3.91**	-4.28**	-1.03
15	Sakha106 × Sakha108	2.99**	2.80**	3.40**	0.43	-1.47	-0.53
16	Sakha106 × IET1444	-1.78**	-4.00**	18.84**	7.30**	1.74	2.24**
17	Sakha106 × WAB1573	4.15**	-0.86	5.46**	0.71	-3.11**	-2.03**
18	Sakha106 × NERICA4	-0.75	-0.30	2.17**	-2.19**	-0.48	-0.27
19	Sakha107 × Sakha108	-4.58**	-3.80**	-2.39**	-4.13**	0.69	-0.09
20	Sakha107 × IET1444	-0.01	1.40**	1.79*	-3.93**	3.90**	6.01
21	Sakha107 × WAB1573	-0.08	-1.13*	20.07**	11.81**	-1.62	-0.93
22	Sakha107 × NERICA4	2.02**	-3.23**	-3.32**	5.25**	-4.06**	-3.17**
23	Sakha108 × IET1444	3.09**	-0.16	5.95**	1.74**	4.72**	4.17**
24	Sakha108 × WAB1573	3.02**	-0.70	-0.77	5.15**	-3.14**	-3.43**
25	Sakha108 × NERICA4	0.12	0.54	-11.83**	-8.75**	-0.24	-1.34*
26	IET444 × WAB1573	-2.41**	-4.50**	-3.59**	3.02**	2.41*	1.00
27	IET444 × NERICA4	-0.31	-2.93**	-2.65**	4.79**	-6.03**	-4.57**
28	WAB1573 × NEICA4	-1.71**	1.54**	12.86**	5.87**	4.11**	-0.18
L.S.D 0.05		1.10	0.88	1.72	1.14	2.17	1.05
L.S.D 0.01		1.47	1.17	2.29	1.52	2.89	1.39

*and ** indicate significance at 0.05 and 0.01 probability levels, respectively. N, normal condition; D, water stress condition.

Cont.

No.	Genotypes	Panicle length		Number of filled grains per panicle		Grain yield per plant (g)	
		N	D	N	D	N	D
1	Giza 178 × Giza179	0.18	0.87*	42.60**	36.57**	-1.63**	-0.21
2	Giza178 × Sakha106	-1.86**	-1.69**	2.84	0.63	-1.55**	-1.68**
3	Giza178 × Sakha107	1.28**	2.45**	-2.63	-9.30**	1.47**	2.02**
4	Giza178 × Sakha108	1.50**	1.19**	2.16	-2.40*	3.23**	3.25**
5	Giza178 × IET1444	1.40**	1.36**	14.86**	16.63**	1.72**	0.55
6	Giza178 × WAB1573	-2.56**	-3.07**	21.20**	17.17**	-2.13**	-2.45**
7	Giza178 × NERICA4	2.68**	1.59**	11.04**	-1.97	0.94**	2.82**
8	Giza178 × Giza179	2.20**	1.57**	6.26	-1.47	-0.40	0.92*
9	Giza179 × Sakha107	-0.59	-0.96*	-2.20	-3.73**	4.46**	2.95**
10	Giza179 × Sakha108	0.04	0.18	2.25	2.17	2.99**	3.52**
11	Giza179 × IET1444	-1.63**	-1.32**	17.84**	20.53**	-2.89**	0.82
12	Giza179 × WAB1573	0.90*	-1.32**	12.04**	-1.60	1.00	-3.51**
13	Giza179 × NERICA4	1.91**	0.58	-3.41	-0.73	2.60**	-0.91*
14	Sakha106 × Sakha107	2.36**	1.83**	-2.80	-0.67	1.11**	-1.51**
15	Sakha106 × Sakha108	0.96*	0.30	-0.01	3.57**	0.13	1.05*
16	Sakha106 × IET1444	1.19**	1.80**	-11.64	-15.40**	0.39*	0.35
17	Sakha106 × WAB1573	-1.31**	-0.89*	2.03	8.80**	-3.02**	-1.31**
18	Sakha106 × NERICA4	-1.57**	-1.30**	8.33	12.00**	-0.85**	0.29
19	Sakha107 × Sakha108	3.59**	3.38**	-4.81	-4.70**	0.29	1.42**
20	Sakha107 × IET1444	2.09**	2.54**	2.56	2.33*	5.48**	2.05**
21	Sakha107 × WAB1573	2.66**	2.85**	10.23*	9.53**	-1.47**	0.39
22	Sakha107 × NERICA4	-2.33**	-1.89**	19.53**	20.40**	-3.80**	-1.68**
23	Sakha108 × IET1444	-0.31	1.02*	13.68**	13.90**	7.64**	2.29**
24	Sakha108 × WAB1573	-2.75**	-2.34**	10.68*	11.43**	2.26**	2.95**
25	Sakha108 × NERICA4	-2.74**	-1.42**	10.65*	10.97**	-0.57**	-1.11*
26	IET1444 × WAB1573	4.75**	4.16**	-14.28**	-11.20**	2.32*	0.59
27	IET1444 × NERICA4	-2.57**	-2.58**	-10.98*	-5.33**	-3.05**	-3.81**
28	WAB1573 × NEICA4	3.00**	3.72**	-2.31	7.87**	0.77**	0.19
L.S.D 0.05		0.85	0.81	8.73	2.19	0.42	0.88
L.S.D 0.01		1.13	1.08	11.62	2.91	0.55	1.18

*and ** indicate significance at 0.05 and 0.01 probability levels, respectively. N, normal condition; D, water stress condition.

Supplementary Table S3. Values of 22 stress screening indices based on grain yield under normal or water stress conditions.

Genotype	Yns	Ys	MP	GMP	HM	STI	YI	MSTI1	MSTI2	YSI	RSI	DI
G1	44.53	34.67	39.600	231.356	38.986	0.822	1.020	0.869	0.856	0.779	0.993	0.794
G2	46.83	37.67	42.250	257.785	41.754	0.940	1.109	1.098	1.155	0.804	1.026	0.892
G3	41.83	32.33	37.080	209.098	36.472	0.720	0.951	0.671	0.652	0.773	0.985	0.735
G4	47.30	38.33	42.815	263.615	42.345	0.966	1.128	1.151	1.229	0.810	1.033	0.914
G5	52.03	40.00	46.015	288.527	45.229	1.109	1.177	1.599	1.536	0.769	0.980	0.905
G6	46.80	36.67	41.735	250.861	41.120	0.914	1.079	1.066	1.065	0.784	0.999	0.846
G7	36.93	30.00	33.465	182.310	33.106	0.590	0.883	0.429	0.460	0.812	1.036	0.717
G8	40.30	35.67	37.985	226.441	37.844	0.766	1.050	0.662	0.844	0.885	1.129	0.929
G9	45.90	36.00	40.950	243.898	40.352	0.880	1.059	0.988	0.988	0.784	1.000	0.831
G10	50.73	38.33	44.530	273.005	43.667	1.036	1.128	1.420	1.318	0.756	0.963	0.852
G11	53.20	40.33	46.765	294.160	45.880	1.143	1.187	1.723	1.610	0.758	0.967	0.900
G12	54.70	41.33	48.015	305.674	47.084	1.204	1.216	1.919	1.781	0.756	0.963	0.919
G13	45.10	38.00	41.550	255.195	41.247	0.913	1.118	0.989	1.142	0.843	1.074	0.942
G14	42.97	30.00	36.485	196.655	35.332	0.687	0.883	0.675	0.535	0.698	0.890	0.616
G15	44.87	33.00	38.935	221.051	38.030	0.789	0.971	0.846	0.744	0.735	0.938	0.714
G16	40.90	31.00	35.950	198.255	35.268	0.675	0.912	0.602	0.562	0.758	0.966	0.691
G17	44.77	32.00	38.385	214.113	37.323	0.763	0.942	0.815	0.677	0.715	0.911	0.673
G18	46.77	35.00	40.885	239.360	40.038	0.872	1.030	1.016	0.925	0.748	0.954	0.771
G19	43.30	33.67	38.485	221.558	37.883	0.777	0.991	0.776	0.762	0.778	0.991	0.770
G20	33.87	28.33	31.100	164.875	30.853	0.511	0.834	0.312	0.355	0.836	1.067	0.697
G21	36.33	30.33	33.330	182.812	33.060	0.587	0.893	0.413	0.468	0.835	1.064	0.745
G22	42.33	33.00	37.665	214.703	37.087	0.744	0.971	0.710	0.702	0.780	0.994	0.757
G23	49.37	37.67	43.520	264.684	42.734	0.991	1.109	1.286	1.217	0.763	0.973	0.846
G24	50.83	37.67	44.250	268.569	43.272	1.020	1.109	1.404	1.253	0.741	0.945	0.822
G25	37.87	32.33	35.100	198.954	34.881	0.652	0.951	0.498	0.590	0.854	1.089	0.812
G26	35.83	30.67	33.250	183.585	33.050	0.585	0.903	0.400	0.477	0.856	1.091	0.773
G27	44.07	30.00	37.035	199.156	35.699	0.704	0.883	0.729	0.549	0.681	0.868	0.601
G28	55.97	38.33	47.150	286.759	45.500	1.143	1.128	1.907	1.454	0.685	0.873	0.772
G29	44.57	35.33	39.950	235.866	39.416	0.839	1.040	0.888	0.907	0.793	1.011	0.824
G30	42.03	31.67	36.850	205.318	36.122	0.709	0.932	0.667	0.616	0.754	0.961	0.702
G31	38.80	34.00	36.400	211.785	36.242	0.703	1.001	0.563	0.703	0.876	1.117	0.877
G32	40.90	32.33	36.615	206.760	36.114	0.704	0.951	0.628	0.638	0.790	1.008	0.752
G33	35.83	28.33	32.080	169.578	31.642	0.541	0.834	0.370	0.376	0.791	1.008	0.659
G34	32.70	29.67	31.185	169.665	31.111	0.517	0.873	0.294	0.394	0.907	1.157	0.792
G35	33.63	28.67	31.150	166.261	30.953	0.514	0.844	0.309	0.366	0.853	1.087	0.719
G36	35.13	31.00	33.065	183.739	32.936	0.580	0.912	0.381	0.483	0.882	1.125	0.805

Yns, yield under normal conditions; Ys, yield under water stress: MP, mean productivity; GMP, geometric mean productivity; HM, Harmonic mean; STI, stress tolerance index; YI, yield index; MSTI1, modified stress tolerance index-I; MSTI2, modified stress tolerance index-II; YSI, yield stability index; RSI, relative stress index; DI, drought index.

Cont.

Genotype	SNPI	REI	MRP	GM	TOL	SSI	SSPI	YR	ATI	MPI	SSSI	SDI
G1	75.475	1.049	2.048	8.032	9.860	1.026	0.114	0.221	303.843	4.930	0.006	0.221
G2	84.949	1.198	2.189	9.225	9.160	0.907	0.106	0.196	301.734	4.580	-0.020	0.196
G3	69.885	0.919	1.917	7.806	9.500	1.053	0.110	0.227	273.993	4.750	0.011	0.227
G4	87.215	1.231	2.220	9.546	8.970	0.879	0.104	0.190	299.545	4.485	-0.026	0.190
G5	86.036	1.414	2.378	7.650	12.030	1.072	0.139	0.231	430.419	6.015	0.015	0.231
G6	80.340	1.166	2.159	8.240	10.130	1.003	0.117	0.216	329.122	5.065	0.001	0.216
G7	68.470	0.752	1.735	9.658	6.930	0.870	0.080	0.188	180.906	3.465	-0.028	0.188
G8	94.404	0.976	1.980	16.408	4.630	0.533	0.053	0.115	137.675	2.315	-0.101	0.115
G9	78.951	1.122	2.119	8.273	9.900	1.000	0.114	0.216	315.618	4.950	0.000	0.216
G10	81.196	1.321	2.299	7.182	12.400	1.133	0.143	0.244	428.837	6.200	0.029	0.244
G11	85.674	1.457	2.415	7.267	12.870	1.121	0.149	0.242	467.539	6.435	0.026	0.242
G12	87.552	1.535	2.479	7.182	13.370	1.133	0.154	0.244	498.571	6.685	0.029	0.244
G13	91.347	1.164	2.159	11.704	7.100	0.730	0.082	0.157	230.519	3.550	-0.058	0.157
G14	60.146	0.876	1.875	5.626	12.970	1.399	0.150	0.302	365.218	6.485	0.086	0.302
G15	68.439	1.006	2.007	6.560	11.870	1.226	0.137	0.265	358.224	5.935	0.049	0.265
G16	65.844	0.861	1.856	7.263	9.900	1.122	0.114	0.242	276.469	4.950	0.026	0.242
G17	65.077	0.973	1.975	6.012	12.770	1.322	0.147	0.285	379.078	6.385	0.070	0.285
G18	73.560	1.112	2.109	6.947	11.770	1.167	0.136	0.252	373.477	5.885	0.036	0.252
G19	73.208	0.990	1.990	7.993	9.630	1.031	0.111	0.222	288.377	4.815	0.007	0.222
G20	67.328	0.652	1.615	11.227	5.540	0.758	0.064	0.164	134.589	2.770	-0.052	0.164
G21	71.874	0.748	1.731	11.110	6.000	0.766	0.069	0.165	156.203	3.000	-0.051	0.165
G22	71.932	0.949	1.948	8.074	9.330	1.022	0.108	0.220	273.484	4.665	0.005	0.220
G23	80.475	1.263	2.248	7.439	11.700	1.099	0.135	0.237	395.717	5.850	0.021	0.237
G24	78.572	1.300	2.282	6.725	13.160	1.200	0.152	0.259	451.630	6.580	0.043	0.259
G25	79.453	0.832	1.825	12.671	5.540	0.678	0.064	0.146	152.030	2.770	-0.069	0.146
G26	75.732	0.746	1.729	12.888	5.160	0.668	0.060	0.144	134.153	2.580	-0.072	0.144
G27	59.326	0.898	1.900	5.264	14.070	1.480	0.162	0.319	401.232	7.035	0.104	0.319
G28	76.035	1.457	2.420	5.346	17.640	1.461	0.204	0.315	640.789	8.820	0.099	0.315
G29	78.355	1.069	2.068	8.647	9.240	0.961	0.107	0.207	287.564	4.620	-0.008	0.207
G30	66.935	0.904	1.902	7.114	10.360	1.143	0.120	0.246	296.437	5.180	0.031	0.246
G31	87.948	0.896	1.896	15.167	4.800	0.573	0.055	0.124	136.731	2.400	-0.092	0.124
G32	71.485	0.898	1.895	8.545	8.570	0.971	0.099	0.210	244.407	4.285	-0.006	0.210
G33	62.659	0.689	1.661	8.555	7.500	0.970	0.087	0.209	187.403	3.750	-0.006	0.209
G34	83.992	0.659	1.628	20.584	3.030	0.430	0.035	0.093	74.019	1.515	-0.123	0.093
G35	70.285	0.655	1.620	12.560	4.960	0.684	0.057	0.147	120.789	2.480	-0.068	0.147
G36	81.461	0.740	1.723	16.012	4.130	0.545	0.048	0.118	106.891	2.065	-0.098	0.118

SNPI, stress/non-stress productivity index; REI, relative efficiency index: MRP, mean relative performance, GM, golden mean; TOT, tolerance index; SSI, stress susceptibility index; SSPI, stress susceptibility percentage index; YR, yield reduction; ATI, abiotic stress tolerance index; MPI, mean productivity index; SSSI, Schnieders stress susceptibility index; SDI, sensitivity drought index.

Supplementary Table S4. Rank of genotypes by 22 indices and yield under normal or water stress along with their average rank.

Genotype	Yns	Ys	MP	GMP	HM	STI	YI	MSTI1	MSTI2	YSI	RSI	DI
G13	13	7	11	10	10	11	7	12	10	8	8	1
G8	26	13	19	16	18	18	13	24	16	2	2	2
G4	8	4	8	8	8	8	4	8	7	12	12	4
G2	9	8	9	9	9	9	8	9	9	13	13	7
G31	27	17	26	21	22	25	17	27	19	4	4	8
G5	4	3	4	3	4	4	3	4	3	23	23	5
G11	3	2	3	2	2	2	2	3	2	25	25	6
G12	2	1	1	1	1	1	1	1	1	27	27	3
G29	16	14	14	14	14	14	14	14	14	14	14	13
G6	10	11	10	11	11	10	11	10	11	18	18	11
G9	12	12	12	12	12	12	12	13	12	17	17	12
G23	7	8	7	7	7	7	8	7	8	24	24	10
G10	6	4	5	5	5	5	4	5	5	28	28	9
G25	28	21	28	26	28	28	21	28	25	6	6	15
G36	33	26	32	29	32	32	26	32	29	3	3	16
G34	36	33	34	33	34	34	33	36	33	1	1	18
G1	17	16	15	15	15	15	16	15	15	20	20	17
G26	31	28	31	30	31	31	28	31	30	5	5	19
G24	5	8	6	6	6	6	8	6	6	31	31	14
G19	19	18	17	17	17	17	18	18	17	21	21	22
G28	1	4	2	4	3	3	4	2	4	35	35	20
G22	21	19	20	19	20	20	19	20	20	19	19	23
G32	24	21	24	23	24	23	21	25	23	16	16	24
G21	30	29	30	31	30	30	29	30	31	10	10	25
G7	29	30	29	32	29	29	30	29	32	11	11	28
G18	11	15	13	13	13	13	15	11	13	30	30	21
G35	35	34	35	35	35	35	34	35	35	7	7	27
G3	23	21	21	22	21	21	21	22	22	22	22	26
G20	34	35	36	36	36	36	35	34	36	9	9	31
G15	14	19	16	18	16	16	19	16	18	32	32	29
G33	31	35	33	34	33	33	35	33	34	15	15	34
G30	22	25	23	24	23	22	25	23	24	29	29	30
G16	24	26	27	27	27	27	26	26	26	26	26	32
G17	15	24	18	20	19	19	24	17	21	33	33	33
G14	20	30	25	28	26	26	30	21	28	34	34	35
G27	18	30	22	25	25	24	30	19	27	36	36	36

Yns, yield under normal conditions; Ys, yield under water stress: MP, mean productivity; GMP, geometric mean productivity; HM, Harmonic mean; STI, stress tolerance index; YI, yield index; MSTI1, modified stress tolerance index-I; MSTI2, modified stress tolerance index-II; YSI, yield stability index; RSI, relative stress index; DI, drought index.

Cont.

Genotype	SNPI	REI	MRP	GM	TOL	SSI	SSPI	YR	ATI	MPI	SSSI	SDI	AR
G13	2	11	11	8	11	8	11	8	12	11	8	8	9
G8	1	18	18	2	3	2	3	2	7	3	2	2	10
G4	5	8	8	12	14	12	14	12	20	14	12	12	10
G2	8	9	9	13	15	13	15	13	21	15	13	13	11
G31	3	25	24	4	4	4	4	4	6	4	4	4	13
G5	6	4	4	23	28	23	28	23	32	28	23	23	14
G11	7	2	3	25	31	25	31	25	34	31	25	25	14
G12	4	1	1	27	34	27	34	27	35	34	27	27	14
G29	17	14	14	14	16	14	16	14	17	16	14	14	15
G6	13	10	10	18	23	18	23	18	24	23	18	18	15
G9	15	12	12	17	21	17	21	17	23	21	17	17	15
G23	12	7	7	24	25	24	25	24	29	25	24	24	16
G10	11	5	5	28	29	28	29	28	31	29	28	28	16
G25	14	28	28	6	7	6	7	6	8	7	6	6	16
G36	10	32	32	3	2	3	2	3	2	2	3	3	16
G34	9	34	34	1	1	1	1	1	1	1	1	1	17
G1	20	15	15	20	20	20	20	20	22	20	20	20	18
G26	19	31	31	5	6	5	6	5	4	6	5	5	18
G24	16	6	6	31	33	31	33	31	33	33	31	31	19
G19	22	17	17	21	19	21	19	21	18	19	21	21	19
G28	18	3	2	35	36	35	36	35	36	36	35	35	19
G22	23	20	20	19	17	19	17	19	14	17	19	19	19
G32	25	23	25	16	13	16	13	16	13	13	16	16	20
G21	24	30	30	10	9	10	9	10	9	9	10	10	20
G7	28	29	29	11	10	11	10	11	10	10	11	11	21
G18	21	13	13	30	26	30	26	30	27	26	30	30	21
G35	26	35	35	7	5	7	5	7	3	5	7	7	21
G3	27	21	21	22	18	22	18	22	15	18	22	22	21
G20	30	36	36	9	7	9	7	9	5	7	9	9	23
G15	29	16	16	32	27	32	27	32	25	27	32	32	24
G33	34	33	33	15	12	15	12	15	11	12	15	15	24
G30	31	22	22	29	24	29	24	29	19	24	29	29	25
G16	32	27	27	26	21	26	21	26	16	21	26	26	26
G17	33	19	19	33	30	33	30	33	28	30	33	33	26
G14	35	26	26	34	32	34	32	34	26	32	34	34	30
G27	36	24	23	36	35	36	35	36	30	35	36	36	30

SNPI, stress/non-stress productivity index; REI, relative efficiency index: MRP, mean relative performance, GM, golden mean; TOT, tolerance index; SSI, stress susceptibility index; SSPI, stress susceptibility percentage index; YR, yield reduction; ATI, abiotic stress tolerance index; MPI, mean productivity index; SSSI, Schnieders stress susceptibility index; SDI, sensitivity drought index; AR, average rank.
